# Pressure overloaded right ventricles: importance of trabeculae in evaluation of RV function by CMR

**DOI:** 10.1186/1532-429X-15-S1-O80

**Published:** 2013-01-30

**Authors:** Mieke Driessen, Tim Leiner, Vivan J Baggen, Hendrik Freling, Petronella Pieper, Folkert J Meijboom, Repke J Snijder, Gertjan T Sieswerda, Tineke P Willems

**Affiliations:** 1 Cardiology, University Medical Center Utrecht, Utrecht, Netherlands; 2 Interuniversity Cardiology Institute of the Netherlands, Utrecht, Netherlands; 3 Radiology, University Medical Center Utrecht, Utrecht, Netherlands; 4 Radiology, University Medical Center Groningen, groningen, Netherlands; 5 Cardiology, University Medical Center Groningen, groningen, Netherlands; 6 Pulmonology, St. Antonius Hospital, Nieuwegein, Netherlands

## Background

Cardiac magnetic resonance imaging (CMR) is the preferred method to evaluate right ventricular (RV) volumes and ejection fraction. In CMR-volumetry, trabeculae and papillary muscles can be either in- or excluded from the blood volume and both methods are used throughout literature. This study aimed to determine the impact of trabeculae and papillary muscles on right ventricular (RV) volumes and function assessed by CMR in different patient groups with pressure overloaded RVs using semi-automatic software and to determine the reproducibility of this method.

## Methods

Four groups of 20 patients (pulmonary hypertension, arterial switch operation (ASO), Tetralogy of Fallot (TOF), systemic RV) and 20 healthy subjects underwent short-axis multislice cine CMR. End diastolic volume (EDV), end systolic volume (ESV), RV mass and ejection fraction (EF) were measured using 2 methods. First, only manual contour tracing of the endo and epicardial borders was performed thus including trabeculae in the blood volume (method 1). With method 2, trabeculae were excluded from the blood volume using semi-automatic pixel-intensity based software. Differences in EDV, ESV volumes, RVEF and RV mass after excluding trabeculae were tested using paired samples T-test. For intra- and interobserver agreement 25 datasets were re-analyzed.

## Results

Exclusion of trabeculae from the blood volume resulted in diminished EDV and ESV and significantly increased RVEF and RV mass (table). The differences were significantly larger for all patient groups compared to healthy controls (P<0.01). For both methods there was a high inter- and intraboserver agreement in all measurements (ICC >0.9 for all measurements). The difference in RVEF between first and second observer were equal for both methods (respectively 0.9 ± 2.6 and -0.5 ± 2.6). For EDV, ESV and RV mass exclusion of trabeculae resulted in smaller differences between observers.

**Table 1:** Differences in RV volume and function

		PH (mean±SD)	ASO (mean±SD)	TOF (mean±SD)	Atrial switch (mean±SD)	Controls (mean±SD)
RVEDV (ml/m^2^)	Method 1 Method 2	117.4 ± 31.8 105.1 ± 28.4*	98.7 ± 22.6 87.8 ± 20.4*	98.7 ± 22.6 87.8 ± 20.4*	139.9 ± 33.6 110.7 ± 28.7*	96.9 ± 18.9 91.2 ± 17.8*

RVESV (ml/m^2^)	Method 1 Method 2	75.4 ± 30.0 62.7 ± 25.9*	49.4 ± 12.9 38.8 ± 10.8*	85.5 ± 27.8 63.7 ± 23.2*	85.9 ± 26.2 57.1 ± 21.7*	47.5 ± 11.5 41.6 ± 10.5*

SV (ml/m^2^)	Method 1 Method 2	42.0 ± 7.9 42.4 ± 8.0**	49.3 ± 11.7 49.1 ± 11.5	61.5 ± 19.4 61.0 ± 19.6**	54.0 ± 14.9 53.6 ± 14.7	49.4 ± 8.6 49.6 ± 8.6

RV mass (gr/m^2^)	Method 1 Method 2	18.5 ± 5.5 31.4 ± 9.8*	19.1 ± 4.8 30.5 ± 7.0*	25.4 ± 7.1 48.7 ± 12.3*	43.3 ± 9.1 73.9 ± 15.4*	13.0 ± 3.0 19.0 4.2*

RVEF (%)	Method 1 Method 2	37.2 ± 8.5 41.9 ± 9.1*	50.0 ± 5.0 56.0 ± 5.2*	42.1 ± 6.9 49.4 ± 12.3*	39.2 ± 7.8 49.3 ± 9.7*	51.3 ± 3.8 54.7 ± 4.1*

Difference in RV volumes and function after exclusion of trabeculae from the blood volume. All volumetric data are indexed for body surface area. *p < 0,001 using paired Student's T-test; ** p < 0.05 using paired Student's T-test

## Conclusions

Exclusion of trabeculae and papillary muscles from the blood volume results in a significant and clinically relevant change in RV volumes, RVEF and RV mass, for patients with pressure overloaded RVs. Using semi-automatic pixel-intensity based software, exclusion of trabeculae from the blood volume is highly reproducible.

## Funding

Interuniversity Cardiology Institute of the Netherlands.

Department of Radiology, University Medical Center Utrecht.

